# Channel identification machines for multidimensional receptive fields

**DOI:** 10.3389/fncom.2014.00117

**Published:** 2014-09-26

**Authors:** Aurel A. Lazar, Yevgeniy B. Slutskiy

**Affiliations:** Bionet Group, Department of Electrical Engineering, Columbia University in the City of New YorkNew York, NY, USA

**Keywords:** channel identification machines, spiking neural circuits, biophysical neuron models, system identification, multidimensional receptive fields, RKHS, time encoding

## Abstract

We present algorithms for identifying multidimensional receptive fields directly from spike trains produced by biophysically-grounded neuron models. We demonstrate that only the projection of a receptive field onto the input stimulus space may be perfectly identified and derive conditions under which this identification is possible. We also provide detailed examples of identification of neural circuits incorporating spatiotemporal and spectrotemporal receptive fields.

## 1. Introduction

Recently, a novel class of system identification algorithms, called Channel Identification Machines (CIMs), has been developed for identifying dendritic processing in spiking neural circuits (Lazar and Slutskiy, 2010, 2012). In the simplest setting, CIMs allow one to identify a communication/processing channel in [Filter]-[Asynchronous Sampler] circuits, where the effect of the channel on an input signal can be described by a *linear* filter and the output of the channel is mapped, or encoded, into a time sequence by a non-linear asynchronous sampler. Such [Filter]-[Asynchronous Sampler] circuits are known in the literature as Time Encoding Machines (TEMs) and their specific embodiments in neuroscience include neural circuit models in which an analog dendritic stimulus processor is followed by a point neuron model encoding the aggregate dendritic current produced by the processor. A few examples of asynchronous samplers include asynchronous sigma/delta modulators (ASDMs), conductance-based point neuron models such as Hodgkin-Huxley, Morris-Lecar, Fitzhugh-Nagumo, Wang-Buzsáki, Hindmarsh-Rose, oscillators with multiplicative coupling and simpler models such as the integrate-and-fire neuron or the threshold-and-fire neuron (Brown et al., 2004; Lazar and Slutskiy, 2012, 2014a).

The above-mentioned asynchronous samplers incorporate the temporal dynamics of spike generation at the axon hillock of biological neurons and allow one to consider, in particular for neuroscience applications, more biologically-plausible non-linear spike generation (sampling) mechanisms. This is in contrast to existing methods, such as the generalized linear model (GLM), which typically assumes a simplified description of the spike generation dynamics by using a static non-linearity. The non-linear contribution of a dynamical system such as the Hodgkin-Huxley neuron model is not static, but dynamic and stimulus-driven (Lazar and Slutskiy, 2014a). It changes from one spike to the next and thus affects the estimation procedure if not properly taken into account. Furthermore, since the non-linearity does not fully capture the complex spike generation dynamics, the filters fit to that non-linearity may not provide an adequate description of the neural circuit, and in particular may confound dendritic processing and encoding (Lazar and Slutskiy, 2014a).

More recently, the CIM methodology has been extended to neural circuits in higher brain centers that receive multidimensional spike trains as input stimuli instead of continuous signals and to circuits with extensive lateral connections and feedback (Lazar and Slutskiy, 2014a). Together with TEMs and Time Decoding Machines (TDMs) for decoding stimuli from spike trains, CIMs provide a unified framework for studying encoding, decoding and identification in neural systems.

The motivation for multidimensional CIMs (Lazar and Slutskiy, 2013) is provided by the concept of a receptive field that is well established in neuroscience. Introduced in 1906 by Sherrington (Sherrington, 1906) to describe an area of the body surface capable of eliciting a reflex in response to a stimulus, the term “receptive field” has been extended to many different sensory modalities and spans many different types of neurons. For example, in the visual system, the receptive field of a photoreceptor is a 3-dimensional cone in space comprising all possible directions in which light can hit the photoreceptor. In the auditory system, receptive fields can correspond to certain spectral regions of audio stimuli. More broadly, the receptive field is that part of the the sensory space that can evoke a neuronal response (Dayan and Abbott, 2005). Spatial and spatiotemporal receptive fields have been successfully used in vision to model retinal ganglion cells in the retina as well as neurons in the lateral geniculate nucleus and the visual cortex (see DeAngelis et al., 1995 for a review). Similarly, spectrotemporal receptive fields have been used to describe responses of auditory neurons (Aertsen and Johannesma, 1981), neurons in cochlear nuclei (Clopton and Backoff, 1991) and neurons in the auditory cortex (Kowalski et al., 1996).

Circuits that process multidimensional feedforward input are often encountered in biological systems (e.g., the retina, Pillow et al., 2008). From a modeling standpoint, one can also consider spiking neural circuits in which, in addition to feedforward input, every neuron may receive lateral inputs from neurons in the same layer. Furthermore, back-propagating action potentials (Waters et al., 2005), or feedback, may affect computations performed within the dendritic tree. Until now, CIMs for circuits with lateral connectivity and feedback have only been considered in the context of scalar or vector-valued signals of one variable, e.g., functions of time *u*_1_(*t*), *t* ∈ ℝ.

In this paper we discuss multidimensional channel identification machines that allow one to identify signal transformations applied to multidimensional signals *u*_*n*_(*x*_1_, …, *x*_*n*_), *n* ∈ ℕ, where *x*_*n*_ typically designates the time variable. A few examples of multidimensional CIMs include (i) spatial CIMs, where the input signal *u*_2_(*x, y*) is a function of a two-dimensional space, describing, e.g., images; (ii) spectrotemporal CIMs, where the input signal *u*_2_(ν, *t*) is a function of spectrum and time, describing, e.g., auditory signals; (iii) spatiotemporal CIMs, where the input signal *u*_3_(*x, y, t*) is a function of space and time, describing, e.g., video signals. Signal transformations are performed by dendritic stimulus processors. They are modeled here as receptive fields whose output is encoded by spiking neurons with lateral connectivity (cross-feedback) and feedback.

The rigorous mathematical formalism employed here enables us to obtain two key results: (i) the projection of the multidimensional receptive field onto the input signal space can be perfectly identified, and (ii) the problem of identification of multidimensional receptive fields is dual to the problem of neural decoding. The duality result shows that the identification of a multidimensional receptive field is equivalent to the problem of encoding its projection with a neural circuit whose receptive field has an impulse response that is exactly the multidimensional input test signal. We provide an identification algorithm and give detailed examples arising in spiking models of audition and vision.

## 2. Methods

### 2.1. Modeling multidimensional stimuli and their processing

#### 2.1.1. The space of input stimuli

**Figure 1:**
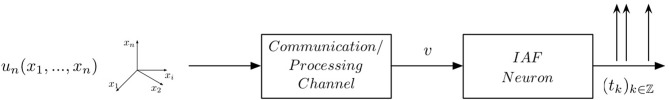
**Multidimensional problem setting**. A known multidimensional signal *u*_*n*_(*x*_1_, *x*_2_, …, *x*_*n*_), is first passed through a communication/processing channel. A non-linear sampler then maps the output *v* of the channel into an observable time sequence (*t*_*k*_)_*k*∈ℤ_.

A multidimensional communication/processing channel of interest is shown in Figure 1. An analog signal *u*_*n*_(*x*_1_, …, *x*_*n*_) of *n* dimensions is passed through a channel with memory that describes a physical communication link or a signal processing stage. The output of the channel *v* is then mapped, or encoded, by an asynchronous sampler into the time sequence (*t*_*k*_)_*k*∈ℤ_. In the example shown in Figure 1, the asynchronous sampler is a (leaky) IAF neuron.

We model input signals as elements of a Reproducing Kernel Hilbert Space (RKHS) (Berlinet and Thomas-Agnan, 2004). For practical and computational reasons we choose to work with the multidimensional space of trigonometric polynomials 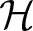
_*n*_ defined below. However, the results are not limited to this particular RKHS.

**Definition 1**. *The space of trigonometric polynomials 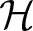
_n_ is a Hilbert space of complex-valued functions u_n_ = u_n_* (*x_1_, …, x_n_*)*, where*

$$u_{n}(x_{1},\ldots ,x_{n})=\sum_{l_{1}\;=\;-L_{1}}^{L_{1}}\ldots \sum_{l_{n}\;=\;-L_{n}}^{L_{n}}u_{l_{1}\ldots l_{n}}e_{l_{1}\ldots l_{n}}(x_{1},\ldots ,x_{n}),$$

*defined over the domain 𝔻_n_* = [*0, T*_1_] × [*0, T*_2_] × ··· × [*0, T_n_*], *where u*_*l*_1_…*l*_*n*__ ∈ ℂ *and*

$$e_{l_{1}\ldots l_{n}}(x_{1},\ldots ,x_{n})=\frac{1}{\sqrt{T_{1}\cdots T_{n}}}\exp\text{​}\left(\frac{jl_{1}\Omega_{1}x_{1}}{L_{1}}+\ldots +\frac{jl_{n}\Omega_{n}x_{n}}{L_{n}}\right)\text{​}.$$

*Here Ω_i_ is the bandwidth in dimension x_i_, *L*_i_ is the order, and *T*_i_* = 2π*L_i_/Ω_i_ is the period, for all *i* = 1, …, n, and 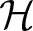
_n_ is endowed with the inner product* 〈·,·〉 : 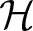
*_n_ × 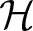
_n_ → ℂ*

(1) $$\langle u_{n},w_{n}\rangle =\int_{{𝔻}_{n}}u_{n}(x_{1},\ldots ,x_{n})\bar{w_{n}(x_{1},\ldots ,x_{n})}dx_{1}\ldots dx_{n}.$$

Note that given the inner product in (1), the set of elements *e*_*l*_1_ … *l*_*n*__(*x*_1_, …, *x*_*n*_) forms an orthonormal basis in 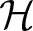
_*n*_. Moreover, 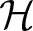
_*n*_ is an RKHS with a reproducing kernel (RK) given by

$$\begin{array}{l} K_{n}(y_{1},\ldots ,y_{n};x_{1},\ldots ,x_{n})=\\ \sum_{l_{1}\;=\;-L_{1}}^{L_{1}}\text{​}\text{​​}\ldots \text{​​​}\sum_{l_{n}\;=\;-L_{n}}^{L_{n}}e_{l_{1}\ldots l_{n}}(y_{1},\ldots ,y_{n})\;\bar{e_{l_{1}\ldots l_{n}}(x_{1},\ldots ,x_{n})}. \end{array}$$

In what follows we provide two- and three-dimensional signal models that arise in audition and vision. For convenience, we use a simpler and widely-used notation.

**Example 1**. *We model spectrotemporal stimuli u*_2_(*ν, t*) *as elements of an RKHS of trigonometric polynomials* 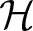
_2_ *defined on* 𝔻_2_ = [*0, T*_1_] × [*0, T*_2_], *where T*_1_ = 2π*L*_1_/Ω_1_, *T*_2_ = 2π*L*_2_/Ω_2_, *with* (Ω_1_, *L*_1_) *and* (Ω_2_, *L*_2_), *being the (bandwidth, order) pairs in the spectral direction ν and in time t, respectively. A signal u*_2_ ∈ 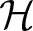
_2_ *can be written as*

$$u_{2}(\nu ,t)=\sum_{|l_{1}|\;\leq \;L_{1}}\sum_{|l_{2}|\;\leq \;L_{2}}u_{l_{1}l_{2}}e_{l_{1}l_{2}}(\nu ,\;t),\;(\nu ,\;t)\in {𝔻}_{2},$$

*where the coefficients u*_*l*_1_*l*_2__ ∈ ℂ *and functions*

$$e_{l_{1}l_{2}}(\nu ,\;t)=\frac{1}{\sqrt{T_{1}T_{2}}}\exp\left(\frac{jl_{1}\Omega_{1}\nu }{L_{1}}+\frac{jl_{2}\Omega_{2}t}{L_{2}}\right)$$

*form an orthonormal basis for the* (2*L*_1_ + 1)(2*L*_2_ + 1)-*dimensional space* 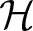
_2_.

**Example 2**. *We model spatiotemporal stimuli u*_3_(*x, y, t*) *as elements of an RKHS of trigonometric polynomials* 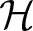
_3_ *defined on* 𝔻_3_ = [*0, T*_1_] × [*0, T*_2_] × [*0, T*_3_], *where T*_1_ = 2π*L*_1_/Ω_1_, *T*_2_ = 2π*L*_2_/Ω_2_, *T*_3_ = 2π*L*_3_/Ω_3_, *with* (Ω_1_, *L*_1_), (Ω_2_, *L*_2_), *and* (Ω_3_, *L*_3_), *being the (bandwidth, order) pairs in spatial directions x and y and in time t, respectively. A visual stimulus u*_3_ ∈ 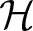
_3_ *can be written as*

$$u_{3}(x,y,t)=\sum_{|l_{1}|\;\leq \;L_{1}}\sum_{|l_{2}|\;\leq \;L_{2}}\sum_{|l_{3}|\;\leq \;L_{3}}u_{l_{1}l_{2}l_{3}}e_{l_{1}l_{2}l_{3}}(x,y,t),\;(x,y,t)\in {𝔻}_{3},$$

*where coefficients u*_*l*_1_*l*_2_*l*_3__ ∈ ℂ *and functions*

$$e_{l_{1}l_{2}l_{3}}(x,y,t)=\frac{1}{\sqrt{T_{1}T_{2}T_{3}}}\exp\left(\frac{jl_{1}\Omega_{1}x}{L_{1}}+\frac{jl_{2}\Omega_{2}y}{L_{2}}+\frac{jl_{3}\Omega_{3}t}{L_{3}}\right)$$

*form an orthonormal basis for the* (2*L*_1_ + 1)(2*L*_2_ + 1)(2*L*_3_ + 1)-*dimensional space* 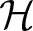
_3_.

#### 2.1.2. Modeling multidimensional processing

In the simplest setting, the communication/processing channel is described by a receptive field with a kernel *h*_*n*_(*x*_1_, …, *x*_*n*_). The kernel is assumed to be causal in the time variable (if any) and BIBO-stable. We also assume that the kernel has a finite support of length *S*_*i*_ ≤ *T*_*i*_ in each direction *x*_*i*_. In other words, each kernel *h*_*n*_ belongs to the space *H*_*n*_.

**Definition 2**. *The filter kernel space *H*_*n*_ is given by*

$$H_{n}=\left\{h_{n}\in L^{1}({\mathbb{R}}^{n})\;|\text{ supp}(h_{n})\subseteq {𝔻}_{n}=[0,T_{1}]\times \cdots \times [0,T_{n}]\right\}.$$

Since the length of the filter support is smaller than or equal to the period of the input signal in each dimension, we effectively require that for given *S*_*i*_ and fixed input signal bandwidth Ω_*i*_, the order *L*_*i*_ of the space 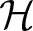
_*n*_ satisfies *L*_*i*_ ≥ *S*_*i*_ · Ω_*i*_/(2π) for all *i* = 1, …, *n*.

**Definition 3**. *The operator 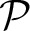: H_n_ → 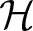
_n_ given (by abuse of notation) by*

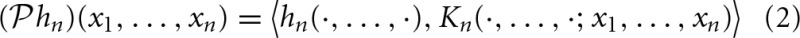



*is called the projection operator*.

Since 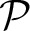
*h*_*n*_ ∈ 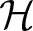
_*n*_, we have

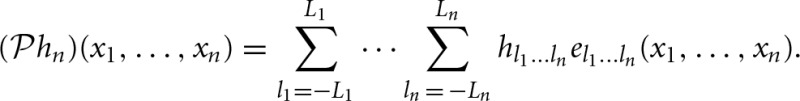



### 2.2. Multidimensional [Filter]-[IAF] tems and their t-transforms

In this section we analyze the general single-input single-output (SISO) multidimensional TEM shown in Figure 1 and describe in detail its I/O behavior. We then provide two specific examples of SISO multidimensional TEMs that are often encountered in neuroscience.

Without loss of generality, we assume that memory effects in the neural circuit arise only in the temporal dimension *t* of the stimulus and interactions in other dimensions are multiplicative in their nature. Consequently, the output *v* of the multidimensional receptive field is given by a convolution in the temporal dimension and integration in all other dimensions, i.e.,

$$\begin{array}{l} v(t)=\int_{{𝔻}_{n}}h_{n}(x_{1},\ldots ,x_{n\;-\;1},s)\\ \;u_{n}(x_{1},\ldots ,x_{n\;-\;1},t-s)dx_{1}\ldots dx_{n\;-\;1}ds. \end{array}$$

The temporal signal *v*(*t*) represents the total dendritic current flowing into the spike initiation zone, where it is encoded into spikes by a point neuron model, such as the (leaky) IAF neuron of Figure 1. The mapping of the multidimensional stimulus *u*_*n*_ into a temporal sequence (*t*_*k*_)_*k*∈ℤ_ is described by the set of equations

(3) $$\int_{t_{k}}^{t_{k+1}}v(t)\exp\left(\frac{t-t_{k\;+\;1}}{RC}\right)dt=q_{k},\;k\in \mathbb{Z},$$

also known as the t-transform (Lazar, 2004; Lazar and Tóth, 2004), where

(4) $$q_{k}=C\delta +bRC\left[\exp\left(\frac{t_{k}-t_{k\;+\;1}}{RC}\right)-1\right].$$

Assuming the stimulus *u*_*n*_(*x*_1_, …, *x*_*n* − 1_, *t*) ∈ 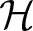
_*n*_ and using the kernel representation, we have

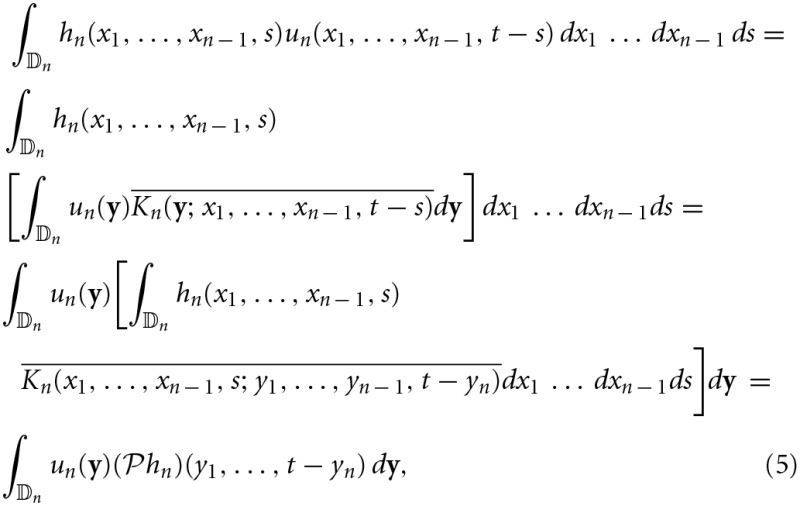



where **y** = (*y*_1_, …, *y*_*n*_) and *d***y** = *dy*_1_ *dy*_2_ … *dy*_*n*_.

Let us now define the linear functional 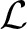
_*k*_ : 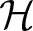
_*n*_ → ℝ

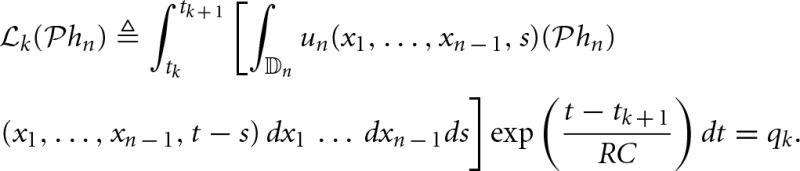



By the Riesz representation theorem there is a function ϕ_*k*_ ∈ 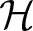
_*n*_ such that

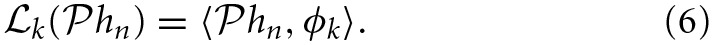



We arrived at the following

**Lemma 1**. *The SISO multidimensional TEM with a receptive field described by a kernel h_n_ = h_n_*(*x*_1_, …, *x*_*n* − 1_, *t*) *provides irregular samples, or quantal measurements of the projection 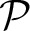
h_n_ of the kernel h_n_ onto the input stimulus space 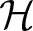
_n_. In other words, the t-transform may be rewritten as an inner product*

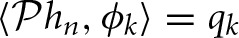



*for every inter-spike interval* [*t_k_, t*_*k* + 1_), *k ∈ ℤ, where ϕ_k_ ∈ 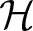
_n_*.

**Remark 1**. *The result above has a simple interpretation. First, information about the receptive field is encoded in the form of quantal measurements q_k_. These measurements can be readily computed from the spike times* (*t_k_*)_*k*∈ℤ_. *Second, the information about the receptive field is only partial and depends on the stimulus space 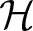
_n_ used in identification. Specifically, q_k_'s are measurements not of the original kernel h_n_ but of its projection 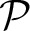
h_n_ onto the space H_n_*.

**Figure 2:**
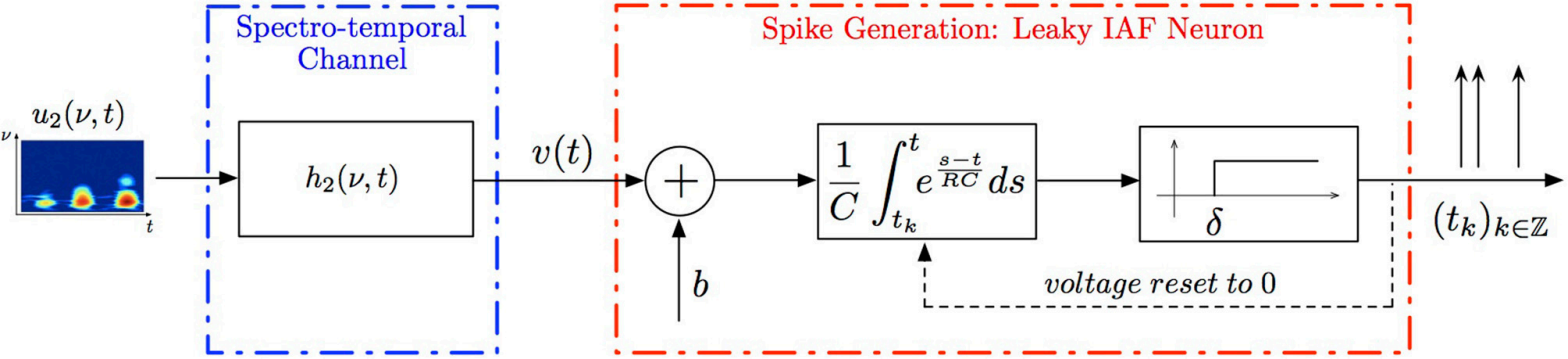
**Block diagram of a circuit with a spectrotemporal communication channel**.

**Example 3**. **A SISO Spectrotemporal TEM** *is shown in Figure 2. The signal u*_2_(ν, *t*), (ν, *t*) ∈ 𝔻_2_ = [*0, T*_1_] × [*0, T*_2_], *is the input to a communication/processing channel with kernel h*_2_(ν, *t*). *The signal u*_2_(ν, *t*) *may represent the time-varying amplitude of a sound in a frequency band centered around ν and h*_2_(ν, *t*) *the spectrotemporal receptive field (STRF) (Kowalski et al., 1996). The output v of the kernel is encoded into a sequence of spike times* (*t_k_*)_*k*∈ℤ_ *by the leaky integrate-and-fire neuron with a threshold δ, bias b and membrane time constant RC. A spectrotemporal TEM can be used to model the processing or transmission of, e.g., auditory stimuli characterized by a frequency spectrum varying in time (Kowalski et al., 1996). The operation of such a TEM can be fully described by the t-transform*

(7) $$\int_{t_{k}}^{t_{k+1}}\left[\int_{{𝔻}_{2}}\text{​}h_{2}(\nu ,s)u_{2}(\nu ,t-s)d\nu ds\right]\exp\text{​}\left(\frac{t-t_{k\;+\;1}}{RC}\right)dt=q_{k},$$

*with q_k_ given by* (4) *for all k ∈ ℤ*.

*Assuming the spectrotemporal stimulus u*_2_(ν, *t*) ∈ 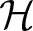
_2_, *Equation (7) can be written as*

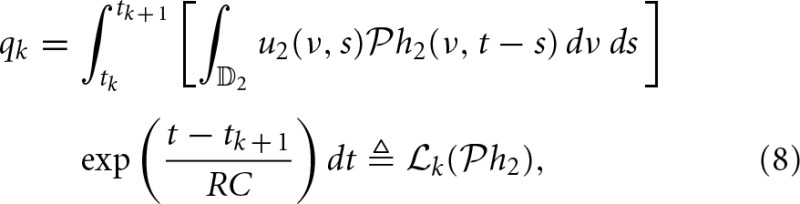



where 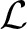
_k_ : 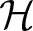
_2_ → ℝ is a linear functional. By the Riesz representation theorem (Berlinet and Thomas-Agnan, 2004), there exists a function ϕ_k_ ∈ 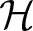
_2_ such that

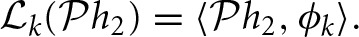



**Figure 3:**
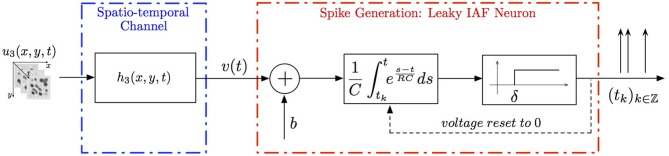
**Block diagram of a circuit with a spatiotemporal communication channel**.

**Example 4**. *A simple* **SISO Spatiotemporal TEM** *is shown in Figure 3. A video signal u*_3_(*x, y, t*), (*x, y, t*) ∈ 𝔻_3_ = [*0, T*_1_] × [*0, T*_2_]| × [*0, T*_3_], *appears as an input to a communication/processing channel described by a filter with a kernel h*_3_(*x, y, t*). *The output v of the kernel is encoded into a sequence of spike times* (*t_k_*)_*k*∈ℤ_ *by the leaky integrate-and-fire neuron*.

A spatiotemporal TEM can be used to model the processing or transmission of, e.g., video stimuli characterized by a spatial component varying in time. The t-transform of such a TEM is given by

(9) $$\begin{array}{l} \int_{t_{k}}^{t_{k+1}}\left[\int_{{𝔻}_{3}}h_{3}(x,y,s)u_{3}(x,y,t-s)dxdyds\right]\\ \;\exp\left(\frac{t-t_{k\;+\;1}}{RC}\right)dt=q_{k}, \end{array}$$

*with q_k_ given by* (4) *for all k ∈ ℤ*.

*Assuming the video stimulus u*_3_(*x, y, t*) ∈ 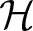
_3_, *Equation (9) can be written as*

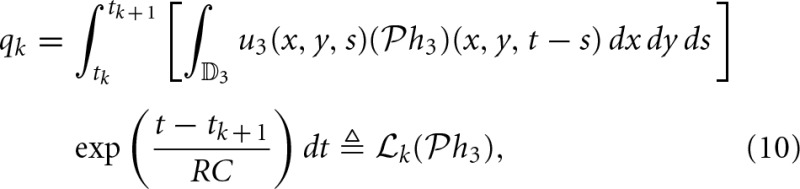



where 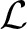
_k_:
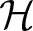
_3_ → ℝ is a linear functional. By the Riesz representation theorem, there is a function ϕ_k_ ∈ 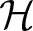
_3_ such that

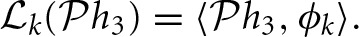



**Example 5**. *A special case of the SISO Spatiotemporal TEM is the* **SISO Spatial TEM**, *in which the communication/processing channel affects only the spatial component of the spatiotemporal input signal. In other words, the output of the receptive field is given by*

$$v(t)=\int_{{𝔻}_{2}}h_{2}(x,y)u_{3}(x,y,t)dxdy.$$

*Note that because only the spatial component of the input is processed, a simpler stimulus that does not vary in time may be presented when identifying this system. For example, such a stimulus may be a static image u*_2_(*x, y*). *Then,*

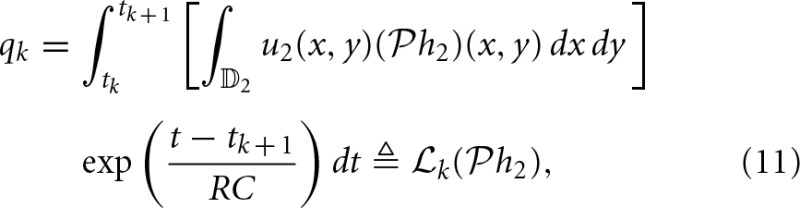



where 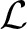
_k_ : 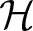
_2_ → ℝ is a functional. As before, by the Riesz representation theorem, there is a function ϕ_k_ ∈ 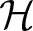
_2_ such that

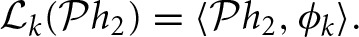



### 2.3. Identification algorithms

In the previous section we demonstrated a relationship between the problem of identification of a receptive field and an irregular sampling problem. Namely, we showed that a projection 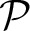
*h*_*n*_ of the multidimensional receptive field *h*_*n*_ is embedded in the output spike sequence of the neuron as samples, or quantal measurements, *q*_*k*_ of 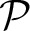
*h*_*n*_. A natural followup question to ask is how to reconstruct 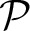
*h*_*n*_ from these measurements. We have the following result.

#### 2.3.1. Feedforward multidimensional SISO CIM

**Theorem 1. (*SISO Multidimensional CIM*)**

*Let* {*u^i^_n_ | u^i^_n_ ∈ 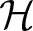
_n_*}^*N*^_*i* = 1_ *be a collection of N linearly independent stimuli at the input to a [Filter]-[Leaky IAF] circuit with a multidimensional receptive field h_n_ ∈ H_n_. If the number of signals N* ≥ ∏^*n* − 1^_*p* = 1_(2*L*_p_ + 1) *and the total number of spikes produced in response to all stimuli is greater than ∏^n^*_*p* = 1_(2*L*_*p*_ + 1) + *N, then the filter projection 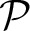
h_n_ can be perfectly identified from a collection of input/output (I/O) pairs* {(*u^i^_n_, 𝕋^i^*)}^*N*^_*i* = 1_ *as*

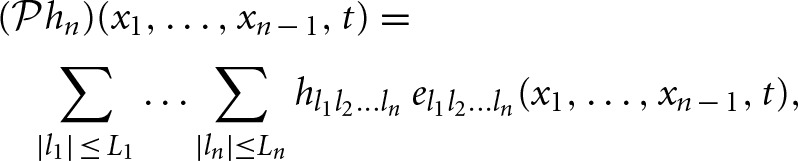



*where* **h** = **Φ**^+^ **q**. *Here* [**h**]_*l*_ = *h*_*l*_1_, …, *l*_*n*__, **Φ** = [**Φ**^1^; **Φ**^2^; …; **Φ**^*N*^] *and the elements of each matrix* **Φ**^*i*^ *are given by*

$$\begin{array}{l} {\left[\Phi^{i}\right]}_{kl}=\frac{RCL_{n}\sqrt{T_{n}}u_{-l_{1},\ldots ,-l_{n-1},l_{n}}^{i}}{jl_{n}\Omega_{n}RC+L_{n}}[e_{l_{n}}\left(t_{k\;+\;1}^{i}\right)\\ \;-e_{l_{n}}\left(t_{k}^{i}\right)\exp\left(\frac{t_{k}^{i}-t_{k\;+\;1}^{i}}{RC}\right)] \end{array}$$

*with the column index l traversing all possible subscript combinations of l*_1_, *l*_2_, …, *l_n_ for all k ∈ ℤ, i* = 1, 2, …, *N. Furthermore*, **q** = [**q**^1^; **q**^2^; …; **q**^*N*^], [**q**^*i*^]_*k*_ = *q*^*i*^_*k*_ *and*

(12) $$q_{k}^{i}=C\delta +bRC\left[\exp\left(\frac{t_{k}^{i}-t_{k\;+\;1}^{i}}{RC}\right)-1\right]$$

*for k ∈ ℤ, i* = 1, …, *N*.

**Proof:** The representation (6) for stimuli *u*^*i*^_*n*_ takes the form

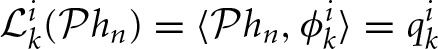



with ϕ^*i*^_*k*_ ∈ 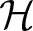
_*n*_. Since 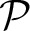
*h*_*n*_ ∈ 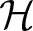
_*n*_ and ϕ^*i*^_*k*_ ∈ 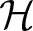
_*n*_, we have

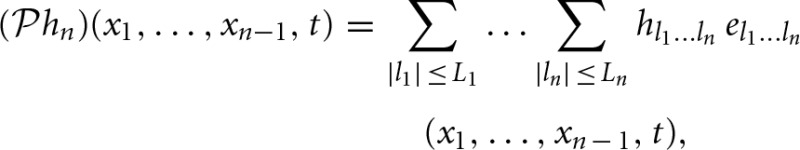



and

$$\begin{array}{l} \phi_{k}^{i}(x_{1},\ldots ,x_{n\;-\;1},t)=\sum_{|l_{1}|\;\leq \;L_{1}}\ldots \sum_{|l_{n}|\;\leq \;L_{n}}\phi_{l_{1}\ldots l_{n}k}^{i}e_{l_{1}\ldots l_{n}}\\ \;(x_{1},\ldots ,x_{n\;-\;1},t), \end{array}$$

and, therefore,

$$q_{k}^{i}=\sum_{|l_{1}|\;\leq \;L_{1}}\ldots \sum_{|l_{n}|\;\leq \;L_{n}}h_{l_{1}\ldots l_{n}}\bar{\phi_{l_{1}\ldots l_{n}k}^{i}}.$$

In matrix form we have **q**^*i*^ = **Φ**^*i*^**h**, with [**q**^*i*^]_*k*_ = *q*^*i*^_*k*_, where the elements [**Φ**^*i*^]_*kl*_ = ϕ^*i*^_*l*_1_ … *l*_*n*_*k*_, with the column index *l* traversing all possible subscript combinations of *l*_1_, *l*_2_, …, *l*_*n*_ and [**h**]_*l*_ = *h*_*l*_1_, …, *l*_*n*__. Repeating for all signals *i* = 1, …, *N*, we obtain **q** = **Φ*****h*** with **q** = [**q**^1^; **q**^2^; …; **q**^*N*^] and **Φ** = [**Φ**^1^; **Φ**^2^; …; **Φ**^*N*^]. This system of linear equations can be solved for **h**, provided that the rank *r*(**Φ**) of the matrix **Φ** satisfies *r*(**Φ**) = ∏^*n*^_*p* = 1_(2*L*_*p*_ + 1). A necessary condition for the latter is that the total number of spikes generated by all *N* neurons is greater or equal to ∏^*n*^_*p* = 1_(2*L*_*p*_ + 1) + *N*. Then **h** = **Φ**^+^**q**, where **Φ**^+^ denotes a pseudoinverse of Φ. To find the coefficients ϕ^*i*^_*l*_1_ … *l*_*n*_*k*_, we note that

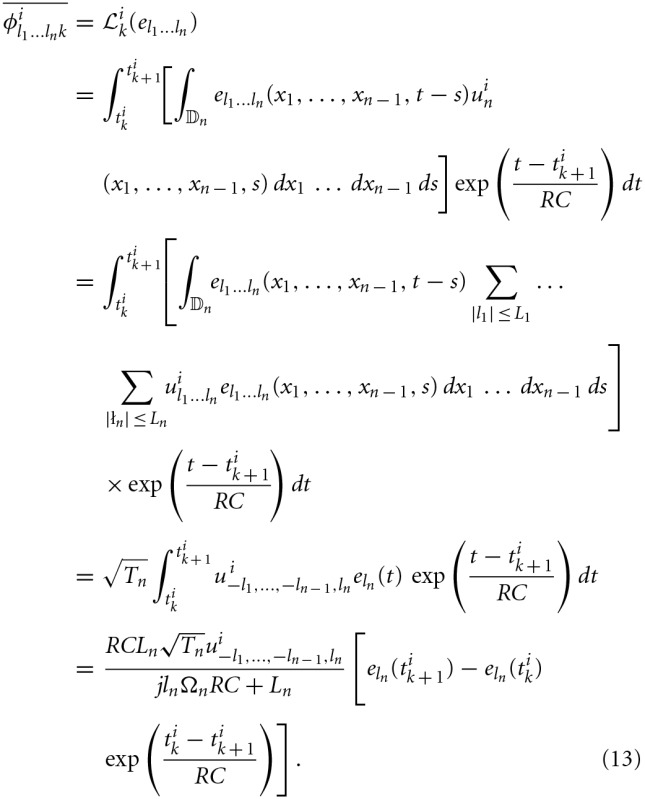



Finally, we note that since the dendritic current *v* has a maximum bandwidth of Ω_*t*_, we only need 2*L*_*t*_ + 1 measurements to specify it. Thus, in response to each stimulus *u*^*i*^_*n*_, the neuron can produce a maximum of only 2*L*_*t*_ + 1 informative measurements, or equivalently, 2*L*_*t*_ + 2 informative spikes on the interval [0, *T*_*t*_]. It follows that if the neuron generates ν ≥ 2*L*_*t*_ + 2 spikes for each test signal, the minimum required number of signals is *N* = ∏^*n*−1^_*p* = 1_(2*L*_*p*_ + 1)(2*L*_*t*_ + 1)/(2*L*_*t*_ + 1) = ∏^*n*−1^_*p* = 1_(2*L*_*p*_ + 1). Similarly, if the neuron generates ν < 2*L*_*t*_ + 2 spikes for each signal, then the minimum required number of signals is $N=\lceil \sum_{p\;=\;1}^{n}\left(2L_{p}+1\right)/(\nu -1)\rceil$.

**Figure 4:**
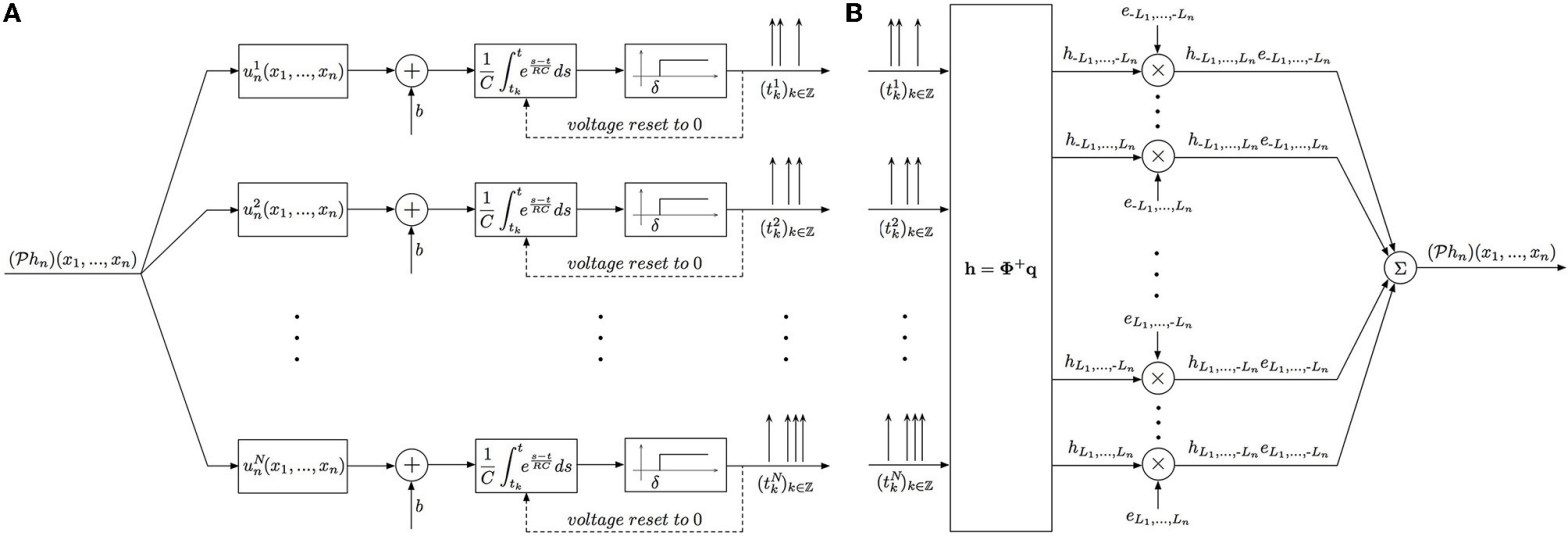
**Block diagram of the Multidimensional CIM**. **(A)** Time encoding interpretation of the multidimensional channel identification problem. **(B)** Block diagram of the multidimensional channel identification machine.

The block diagram of the identification procedure and algorithm are shown in Figure 4. Identification of the filter *h*_*n*_ has been reduced to the encoding of the projection 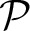
*h*_*n*_ with a SIMO TEM whose receptive fields are *u*^*i*^_*n*_, *i* = 1, …, *N*.

#### 2.3.2. SISO multidimensional CIM with lateral connectivity and feedback

**Figure 5:**
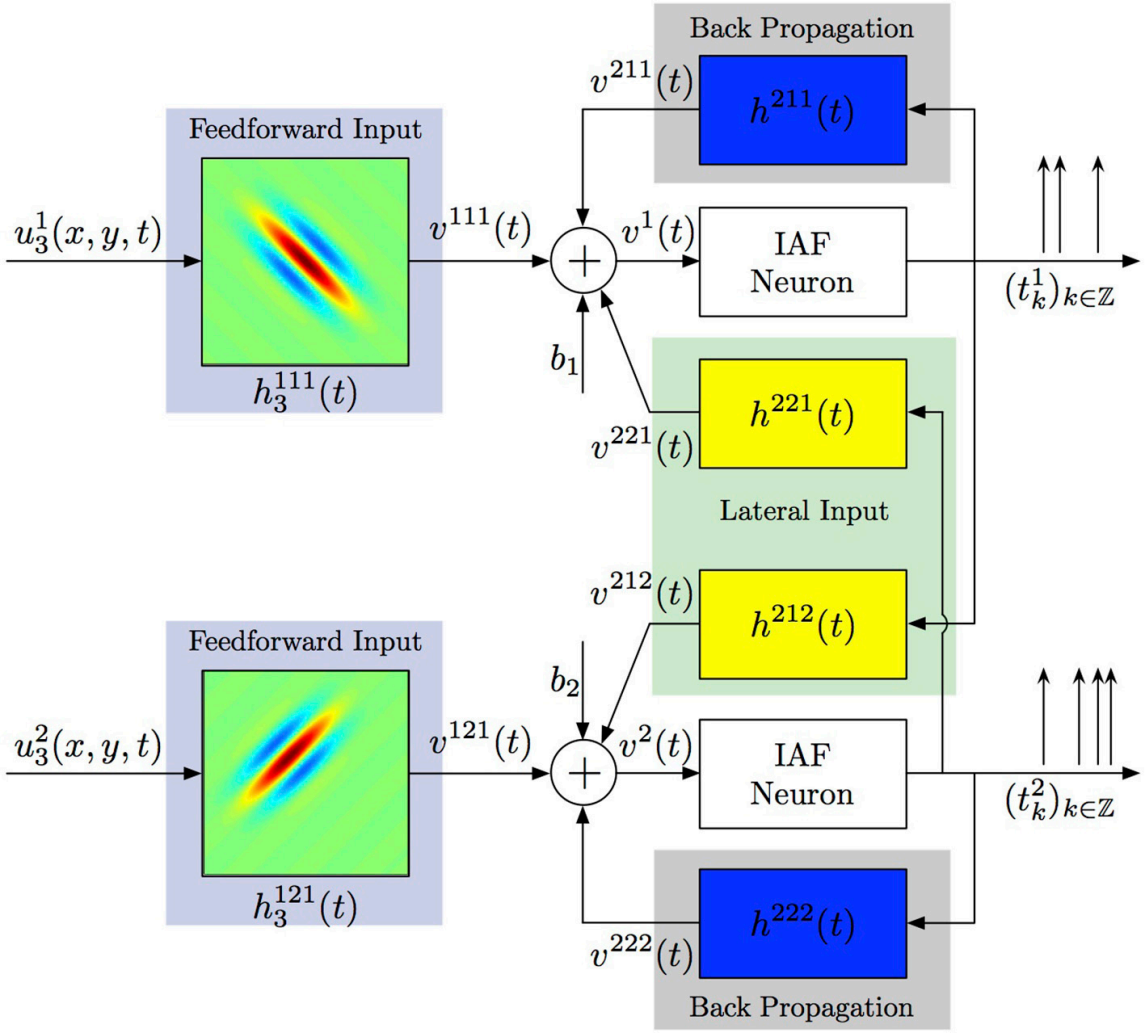
**Identifying spatiotemporal receptive fields in circuits with lateral connectivity and feedback**.

Generalizing the ideas above, one can consider more complex spiking neural circuits, in which every neuron may receive not only feedforward inputs, but also lateral inputs from neurons in the same layer and back-propagating action potentials may contribute to computations within the dendritic tree. A two-neuron circuit incorporating these considerations is shown in Figure 5. Each neuron *j* processes a visual stimulus *u*^*j*^_3_(*x, y, t*) using a distinct spatiotemporal receptive field *h*^1*j*1^_3_(*x, y, t*), *j* = 1, 2. The processing of lateral inputs is described by the temporal receptive fields (cross-feedback filters) *h*^221^ and *h*^212^, while various signals produced by back-propagating action potentials are modeled by the temporal receptive fields (feedback filters) *h*^211^ and *h*^222^. The aggregate dendritic currents *v*^1^ and *v*^2^, produced by the receptive fields and affected by back propagation and cross-feedback, are encoded by IAF neurons into spike times (*t*^1^_*k*_)_*k*∈ℤ_, (*t*^2^_*k*_)_*k*∈ℤ_.

**Theorem 2. (*SISO multidimensional CIM with lateral connectivity and feedback*)**

*Let* {[*u*^1,*i*^_*n*_, *u*^2,*i*^_*n*_] | *u*^*j,i*^_*n*_ ∈ 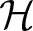
_*n*_, *j* = 1, 2}^*N*^_*i* = 1_ *be a collection of N linearly independent vector stimuli at the input to two neurons with multidimensional receptive fields h*^1*j*1^_*n*_ ∈ *H_n_, j* = 1, 2, *lateral receptive fields h*^212^, *h*^221^ *and feedback receptive fields h*^211^ *and h*^222^. *Let* (*t*^1^_*k*_)_*k*∈ℤ_ *and* (*t*^2^_*k*_)_*k*∈ℤ_ *be the sequences of spike times produced by the two neurons. If the number of test stimuli N* ≥ ∏^*n* − 1^_*p* = 1_(2*L*_*p*_ + 1) + 2 *and the total number of spikes produced by each neuron in response to all stimuli is greater than ∏^n^*_*p* = 1_(2*L*_p_ + 1) + 2(2*L*_*n*_ + 1) + *N, then the filter projections* 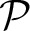
*h*^211^, 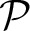
*h*^212^, 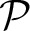
*h*^221^, 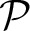
*h*^222^ *and* 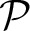
*h*^1*j*1^_*n*_, *j* = 1, 2, *can be identified as* (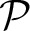
*h*^211^)(*t*) = $\sum_{l\;=\;-L_{n}}^{L_{n}}$ *h*^211^_*l*_ *e*_*l*_(*t*), (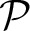
*h*^212^)(*t*) = $\sum_{l\;=\;-L_{n}}^{L_{n}}$ *h*^212^_*l*_ *e*_*l*_(*t*), (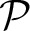
*h*^221^)(*t*) = $\sum_{l\;=\;-L_{n}}^{L_{n}}$ *h*^221^_*l*_ *e*_*l*_(*t*) (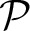
*h*^222^)(*t*) = $\sum_{l\;=\;-L_{n}}^{L_{n}}$ *h*^222^_*l*_ *e*_*l*_(*t*) *and*

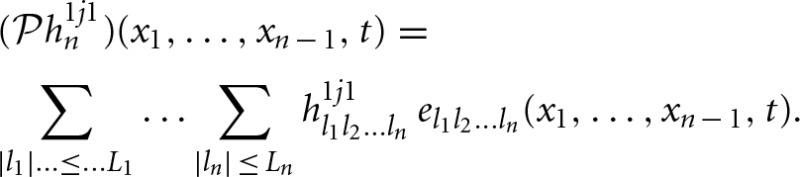



*Here, the coefficients h*^211^_*l*_, *h*^212^_*l*_, *h*^221^_*l*_, *h*^222^_*l*_ *and h*^1*j*1^_*l*_ *are given by* **h** = [**Φ**_*1*_; **Φ**_2_]^+^ **q** *with* **q** = [**q**^11^, …, **q**^1*N*^, **q**^21^, …, **q**^2*N*^]^*T*^, [**q**^*ji*^]_*k*_ = *q*^*ji*^_*k*_ *and* **h** = [**h**^1^; **h**^2^], *where* **h**^*j*^ = [*h*^1*j*1^_−*L*_*n*_, …, −*L*_*n*__, …, *h*^1*j*1^_*L*_*n*_, …, *L*_*n*__, *h*^2[(*j mod* 2) + 1]*j*^_−*L*_, …, *h*^2[(*j mod* 2) + 1]*j*^_*L*_, *h*^2*jj*^_−*L*_, …, *h*^2*jj*^_*L*_]^*T*^, *j* = 1, 2, *provided each matrix* **Φ**_*j*_ *has rank r*(**Φ**_*j*_) = ∏^*n*^_*p* = 1_(2*L*_*p*_ + 1) + 2(2*L*_*n*_ + 1). *The i*^*th*^ *row of* **Φ**_*j*_ *is given by* [**Φ**^1*i*^_*j*_, **Φ**^2*i*^_*j*_, **Φ**^3*i*^_*j*_], *i* = 1, …, *N, with*

$${\left[\Phi_{j}^{2i}\right]}_{kl}=\sqrt{T}\int_{t_{k}^{ji}}^{t_{k+1}^{ji}}t_{l}^{[(j\text{ mod }2)+1]i}e_{l}(t)\exp\left(\frac{t_{k}^{i}-t_{k\;+\;1}^{i}}{RC}\right)dt$$

and

$${\left[\Phi_{j}^{3i}\right]}_{kl}=\sqrt{T}\int_{t_{k}^{ji}}^{t_{k+1}^{ji}}t_{l}^{ji}e_{l}(t)\exp\left(\frac{t_{k}^{i}-t_{k\;+\;1}^{i}}{RC}\right)dt,$$

*l* = −*L*_*n*_, …, *L*_*n*_. *The entries* [**Φ**^1*i*^_*j*_]_*kl*_ *are as given in Theorem 1*.

**Proof:** Essentially the same as in Theorem 1, with an addition of lateral and feedback terms. Each additional temporal filter requires (2*L*_*n*_ + 1) additional measurements, corresponding to the number of bases in the temporal variable *t*.

## 3. Results

**Figure 6:**
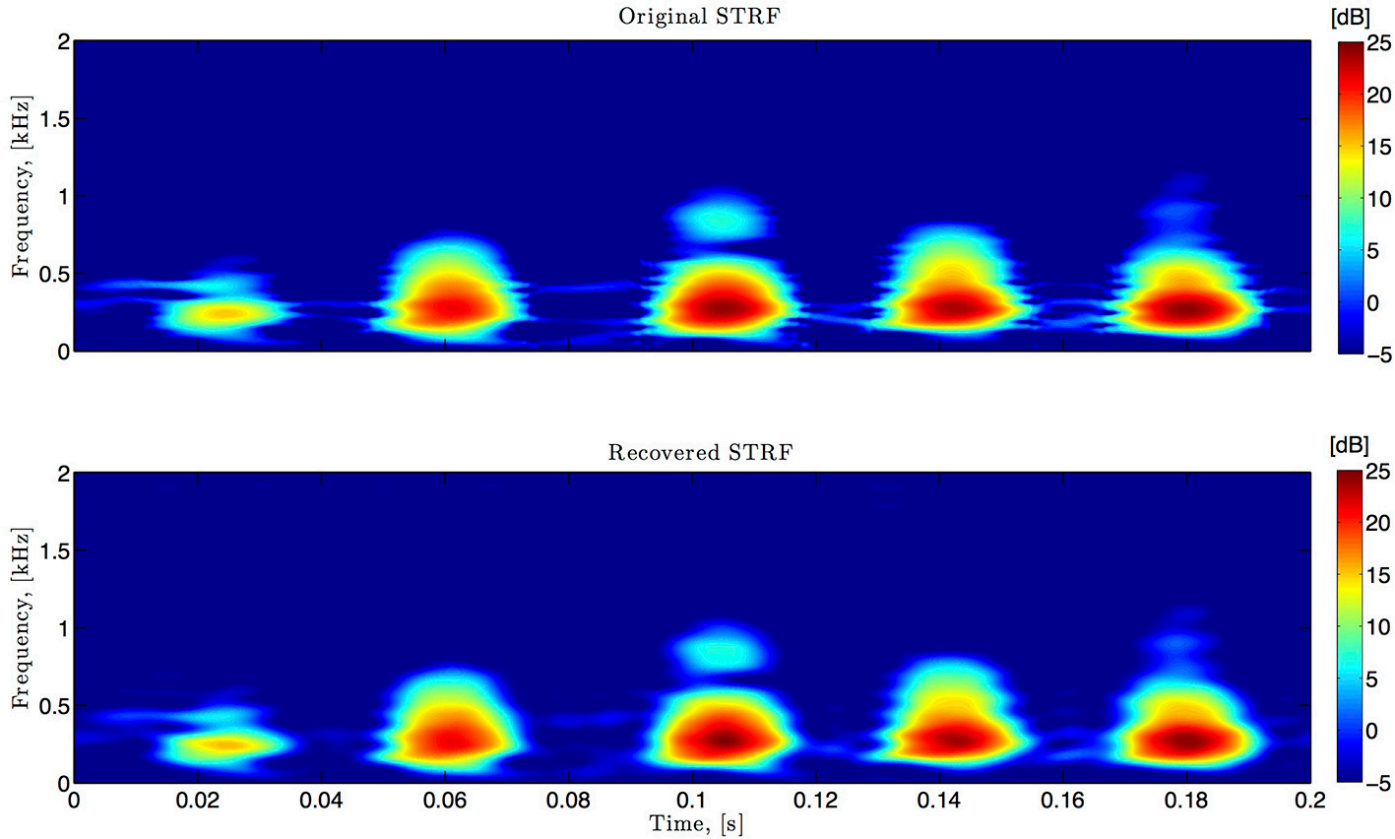
**Spectro-temporal example**. Original and identified spectrotemporal filters are shown in the top and bottom plots, respectively. Ω_1_ = 2π · 80 rad/s, *L*_1_ = 16, Ω_2_ = 2π · 120 rad/s, *L*_2_ = 24.

**Figure 7:**
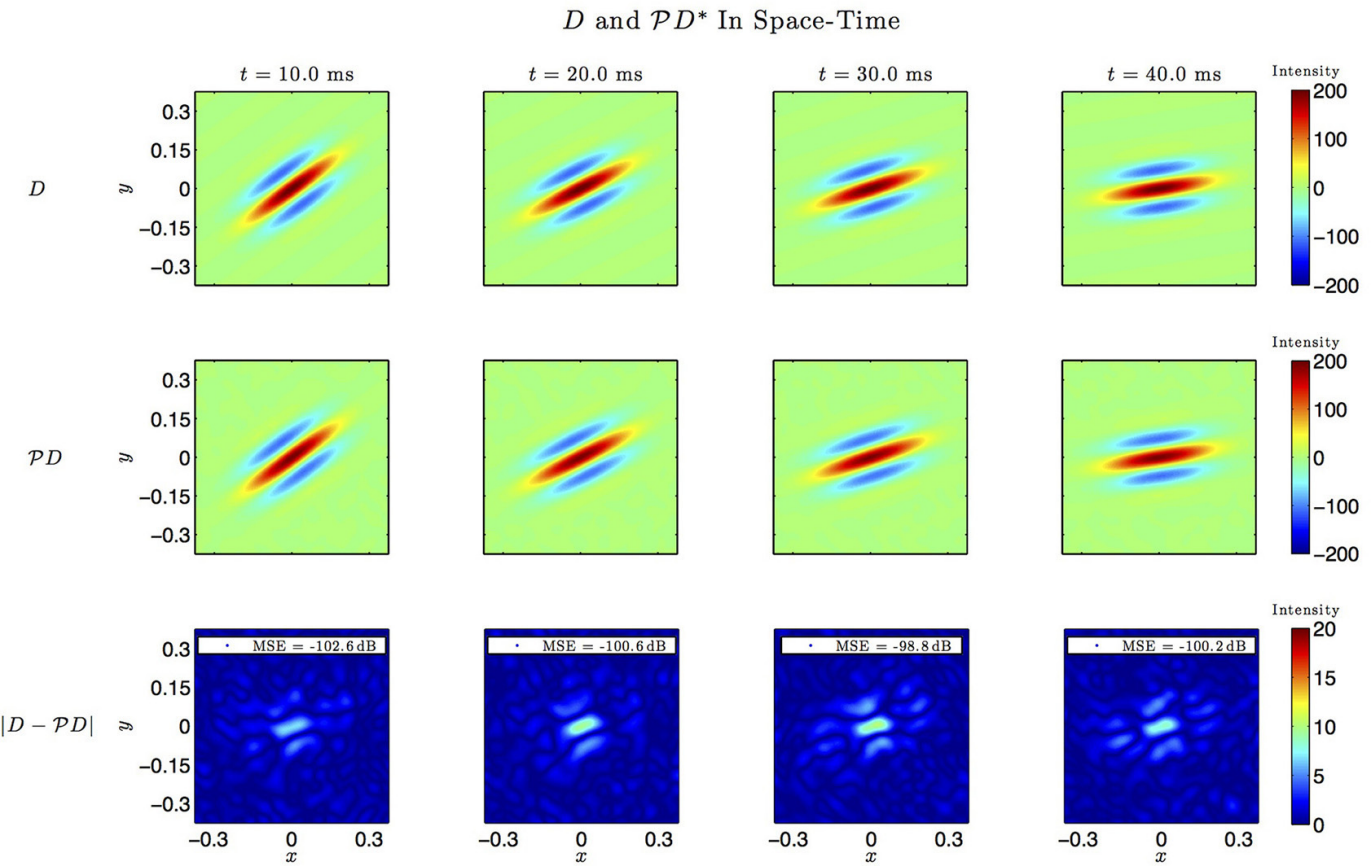
**Spatio-temporal example #1**. **Top row:** Four frames of the original spatiotemporal kernel *h*_3_(*x, y, t*). Here, *h*_3_ is a spatial Gabor function rotating clockwise in space with time. **Middle row:** Four frames of the identified kernel. Ω_1_ = 2π · 12 rad/s, *L*_1_ = 9, Ω_2_ = 2π · 12 rad/s, *L*_2_ = 9, Ω_3_ = 2π · 100 rad/s, *L*_3_ = 5. **Bottom row:** Absolute error between four frames of the original and identified kernel.

**Figure 8:**
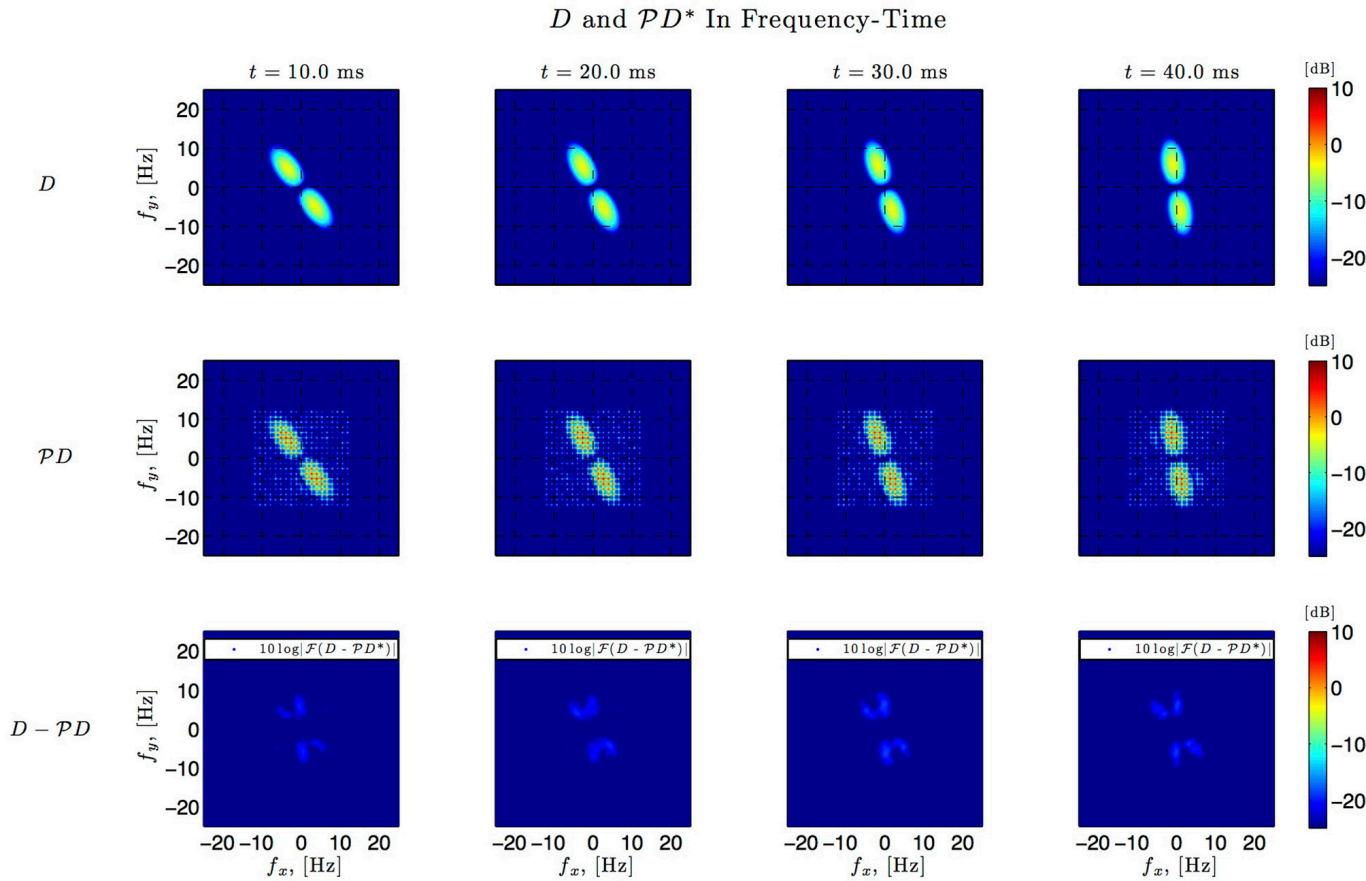
**Spatio-temporal example #1 in the frequency domain**. **Top row:** Fourier amplitude spectrum of the four frames of the original spatiotemporal kernel *h*_3_(*x, y, t*) in Figure 7. Note that the frequency support is roughly confined to a square [−10, 10] × [−10, 10]. **Middle row:** Fourier amplitude spectrum of the four frames of the identified spatiotemporal kernel in Figure 7. Nine spectral lines (*L*_1_ = *L*_2_ = 9) in each spatial direction cover the frequency support of the original kernel. **Bottom row:** Absolute error between four frames of the original and identified kernel.

**Figure 9:**
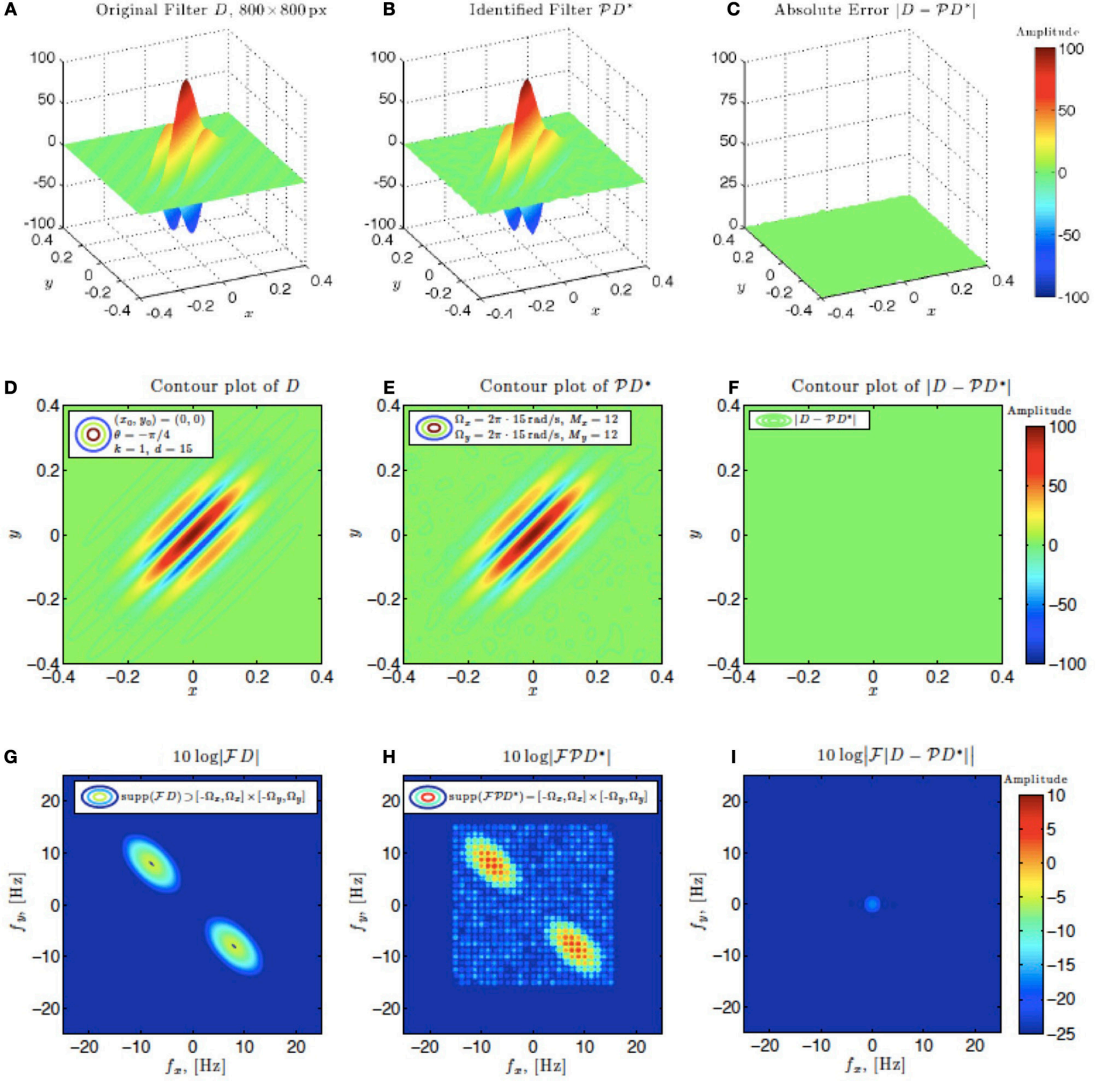
**Spatial example #1**. Ω_1_ = Ω_2_ = 2π · 15 rad/s, *L*_1_ = *L*_2_ = 12. A minimum of *N* = 625 images are required for identification. 1.1 × *N* = 688 images were used. **(A–C)** Left to right: original spatial kernel *h*_2_(*x, y*), identified kernel and absolute error between the two. **(D–F)** Left to right: contour plots of the original spatial kernel *h*_2_(*x, y*), identified kernel and absolute error. **(G–I)** Fourier amplitude spectrum of signals in **(D–E)**.

Figures 6–**9**, and corresponding figure legends demonstrate the performance of the multidimensional Channel Identification Machine.

In simulations pertaining to the spectrotemporal receptive field (see also Figure 6), we used the short-time Fourier transform of an arbitrarily chosen 200 ms segment of the Drosophila courtship song as a model of the STRF. The space of spectrotemporal signals 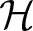
_2_ had bandwidth Ω_1_ = 2π · 80 rad/s and order *L*_1_ = 16 in the spectral direction ν and bandwidth Ω_2_ = 2π · 120 rad/s and order *L*_2_ = 24 in the temporal direction *t*. The STRF appeared in cascade with an ideal IAF neuron (see Figure 2), whose parameters were chosen so that it generated a total of more than (2*L*_1_ + 1)(2*L*_2_ + 1) = 33 × 49 = 1, 617 measurements in response to all test signals. We employed a total of *N* = 40 spectrotemporal signals (which is larger than the (2*L*_1_ + 1) = 33 requirement of Theorem 1) in order to identify the STRF.

In simulations involving the spatiotemporal receptive field (see also Figures 7, 8 we used a spatial Gabor function that was either rotated, dilated or translated in space as a function of time. The space of spatiotemporal signals 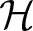
_3_ had bandwidth Ω_1_ = 2π · 12 rad/s and order *L*_1_ = 9 in the spatial direction *x*, bandwidth Ω_2_ = 2π · 12 rad/s and order *L*_2_ = 9 in the spatial direction *y*, and bandwidth Ω_3_ = 2π · 100 rad/s and order *L*_3_ = 5 in the temporal direction *t*. The STRF appeared in cascade with an ideal IAF neuron (see Figure 2), whose parameters were chosen so that it generated a total of more than (2*L*_1_ + 1)(2*L*_2_ + 1)(2*L*_3_ + 1) = 19 × 19 × 11 = 3, 971 measurements in response to all test signals. In order to identify the projection 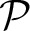
*h*_3_ we employed a total of *N* = 400 spatiotemporal signals, a number that is larger than the (2*L*_1_ + 1)(2*L*_2_ + 1) = 361 requirement of Theorem 1).

In simulations involving the spatial receptive field (see also Figure 9), we used a static spatial Gabor function. The space of spatial signals 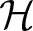
_2_ had bandwidths Ω_1_ = Ω_2_ = 2π · 15 rad/s and *L*_1_ = *L*_2_ = 12 in spatial directions *x* and *y*. The STRF appeared in cascade with an ideal IAF neuron (see Figure 2 as a reference), whose parameters were chosen so that it generated a total of more than (2*L*_1_ + 1)(2*L*_2_ + 1) = 25 × 25 = 625 measurements in response to all test signals. In order to identify the projection 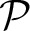
*h*_2_ we employed a total of *N* = 688 spatial signals (a number that is larger than the (2*L*_1_ + 1)(2*L*_2_ + 1) = 625 requirement of Theorem 1).

**Figure 10:**
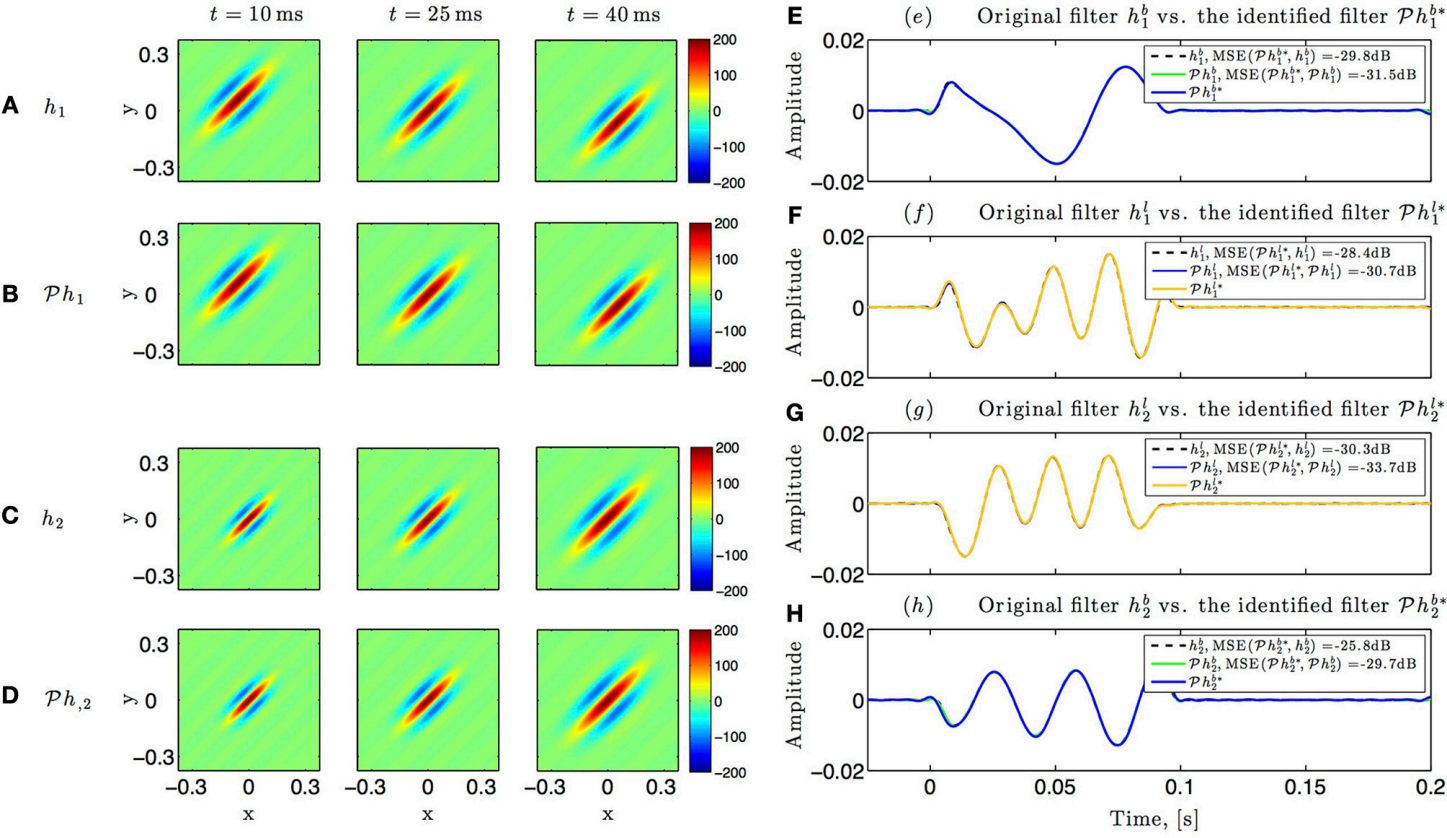
**Identifying spatiotemporal receptive fields in circuits with lateral connectivity and feedback**. **(A–D)** Identifying the feedforward spatiotemporal receptive fields of Figure 5. **(E–H)** Identifying the lateral connectivity and feedback filters of Figure 5.

Identification results for the circuit in Figure 5 are shown in Figure 10. The spatiotemporal receptive fields used in this simulation were non-separable. The first receptive field was modeled as a single spatial Gabor function (at time *t* = 0) translated in space with uniform velocity as a function of time, while the second receptive field was a spatial Gabor function uniformly dilated in space as a function of time. Three different time frames of the original and the identified receptive field of the first neuron are shown in Figures 10A,B, respectively. Similarly, three time frames of the original and identified receptive field of the second neuron are respectively plotted in Figures 10C,D. The identified lateral and feedback kernels are visualized in plots (e-h) of Figure 10.

## 4. Discussion

### 4.1. Implications for multidimensional encoding and decoding

The duality between multidimensional channel identification and stimulus decoding problems allowed us to derive identification algorithms for estimation of receptive fields of arbitrary dimensions and precise conditions under which the identification is possible. At this point it is important to pause and analyze the relationship between the dual problems. As it often turns out, looking at a dual problem can provide a tremendous insight about the primal problem.

Interestingly, previous results for video time encoding and decoding machines provided only the necessary condition of having enough spikes to decode the video (Lazar et al., 2010; Lazar and Pnevmatikakis, 2011). This condition naturally follows from having to invert a matrix in order to compute the basis coefficients of the video signal. Since the matrix needs to be full rank to provide a unique solution, and there are a total of (2*L*_1_ + 1)(2*L*_2_ + 1)(2*L*_3_ + 1) coefficients involved, (2*L*_1_ + 1)(2*L*_2_ + 1)(2*L*_3_ + 1) + *N* spikes are needed from a population of *N* neurons (the number of spikes is larger than the number of needed measurements by *N* since every measurement *q*_*k*_ is computed using two consecutive spikes *t*_*k*_ and *t*_*k* + 1_.).

Note that a necessary condition only tells us that the number of spikes must have been greater than (2*L*_1_ + 1)(2*L*_2_ + 1)(2*L*_3_ + 1) + *N* if we were able to recover the video signal. In order to *guarantee* that the video can be recovered we need a sufficient condition.

The sufficient condition can be derived by drawing comparisons between the decoding and identification problems. In identification, estimation of a receptive field from a single trial is usually not possible, even if the neuron produces a large number of spikes (Lazar and Slutskiy, 2014b). Intuitively, this is because the output of the receptive field is just a function of time. In essence, all dimensions of the stimulus are compressed into just one—the temporal dimension—and we need only (2*L*_3_ + 1) measurements to specify a temporal function. As a result, only (2*L*_3_ + 1) measurements are informative and we do not gain any new information if the neuron is oversampling the temporal signal. Thus, if the neuron is producing at least (2*L*_3_ + 1) measurements per each test stimulus, we need *N* ≥ (2*L*_1_ + 1)(2*L*_2_ + 1) different trials to reconstruct a (2*L*_1_ + 1)(2*L*_2_ + 1)(2*L*_3_ + 1)-dimensional receptive field. Similarly, to decode a (2*L*_1_ + 1)(2*L*_2_ + 1)(2*L*_3_ + 1)-dimensional input stimulus, we need *N* ≥ (2*L*_1_ + 1)(2*L*_2_ + 1) *neurons*, with each neuron in the population producing at least (2*L*_3_ + 1) measurements. If each neuron produces less than (2*L*_3_ + 1) measurements, a larger population *N* is needed to faithfully encode the video signal.

More generally, if the *n*-dimensional input stimulus is an element of a (2*L*_1_ + 1)(2*L*_2_ + 1)…(2*L*_*n*_ + 1)-dimensional RKHS (where the last dimension is time), and the neuron is producing at least at least (2*L*_*n*_ + 1) + 1 spikes per test stimulus, a minimum of (2*L*_1_ + 1)(2*L*_2_ + 1)…(2*L*_*n* − 1_ + 1) linearly independent stimuli, or trials with linearly independent stimuli, are needed to identify the receptive field. This condition is sufficient and by duality between channel identification and time encoding, complements the previous necessary condition derived for time decoding machines.

### 4.2. Extensions

In the derivations above we implicitly assumed that the I/O system was noiseless. In practice, noise is introduced either by the channel or the sampler itself (Lazar et al., 2010). In the presence of noise it is not possible to identify the projection 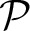
*h*_*n*_ loss-free. However, the analysis/methodology presented above can be extended within an appropriate mathematical setting to I/O systems with noisy measurements. For example, we can still identify an optimal estimate 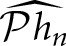 of 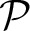
*h*_*n*_ with respect to an appropriately defined cost function, e.g., by using the Tikhonov regularization method. The regularization methodology extensively discussed in Lazar and Slutskiy (2014a) can be adopted with minor modifications to our setting here.

Finally we note that, for convenience and simplicity of notation, the asynchronous sampler used throughout this paper was the IAF neuron. Extensions of our results to neural circuits built with other biophysically-grounded neuron models can be readily obtained by adapting the methodology developed in Lazar and Slutskiy (2012, 2014a).

### Conflict of interest statement

The authors declare that the research was conducted in the absence of any commercial or financial relationships that could be construed as a potential conflict of interest.
